# Translating pathogen biology into diagnostic and management strategies for gummy stem blight in cucumber production

**DOI:** 10.1007/s44297-026-00091-6

**Published:** 2026-11-13

**Authors:** Aastha Ranjan, Alison Lu, Charles Hutton, Oscar Bricknell, Andrew Redman, David T. Tissue, Jonathan M. Plett

**Affiliations:** 1 Hawkesbury Institute for the Environment, Western Sydney University, Locked Bag 1797, Penrith, NSW 2751 Australia; 2 Perfection Fresh Pty Ltd., Level 4, Building F, 1 Homebush Bay Drive, Rhodes, NSW 2138 Australia; 3 Perfection Fresh Pty Ltd., Two Wells Glasshouse Facility, 687 Germantown Rd, Korunye, SA 5502 Australia

**Keywords:** Gummy stem blight, Cucumber, Glasshouse production, Host–pathogen interaction, Effector proteins, Fungicide resistance, Molecular diagnostics, Technology adoption

## Abstract

Gummy stem blight (GSB) is a major disease of cucurbits caused by *Stagonosporopsis* spp. and is increasingly problematic in intensive cucumber production systems, particularly where warm, humid environments favour infection, sporulation and rapid disease spread. GSB can infect vegetative and reproductive tissues and remain latent in fruit for several days before visible symptoms develop, creating a distinctive commercial challenge: disease may be difficult to detect before harvest yet cause significant post-harvest losses, reducing grower and retailer confidence. Compounding these problems, current diagnostic tools for detecting pre-symptomatic infection are often insufficiently validated for species specificity, sensitivity, turnaround time, cost, crude-sample compatibility and practical use by production staff. Many of these limitations reflect the relative lack of genomic resources for *Stagonosporopsis*. Therefore, this review integrates evidence from other pathosystems with current knowledge of *Stagonosporopsis* taxonomy, infection biology, epidemiology, diagnostics, fungicide resistance, effector biology and host resistance to identify pathways towards grower-ready tools. Specifically, we focus on how recent advances in genomics, paired with previously designed detection systems such as loop-mediated isothermal amplification (LAMP) and clustered regularly interspaced short palindromic repeats/CRISPR-associated proteins (CRISPR/Cas), could be adapted for species-specific detection of the causal pathogen and, simultaneously, provide a regional profile of fungicide resistance. In parallel, we explore how a deeper functional genomic understanding of the disease, particularly of effector proteins expressed during the early stages of infection, could provide valuable insights into the dynamics of this host–pathogen interaction and support strategies to disrupt these interactions through novel fungistatic compounds and the development of disease-resistant cultivars. Together, these approaches could support a staged technology-development and adoption roadmap for more precise, rapid and sustainable GSB management in cucumber production.

**Supplementary Information:**

The online version contains supplementary material available at https://doi.org/10.1007/s44297-026-00091-6.

## Cucumber glasshouse production and the industry-wide problem of GSB

Cucumber (*Cucumis sativus*) is the third most widely cultivated vegetable in the world, after tomatoes and onions [1]. Belonging to the same genus of other economically important crops (e.g., *Cucumis melo*), cucumbers are typically cultivated under warm, humid conditions with ample water because they are sensitive to cold and water stress [2]. Although cucumber is grown in both field and glasshouse systems, glasshouses are increasingly favoured because they provide a more controlled environment [3] and higher yield per unit of input than conventional farming systems [4]. Cucumber fruits are highly enriched in water and vitamins A and C, while their skin contains high levels of calcium, potassium and magnesium [5]. Global cucumber production reached 97.8 million metric tonnes in 2023, with volumes projected to reach 115 million metric tonnes by 2035 and market value expected to approach €88.27 billion, rising at 1.8% annually [6]. This increasing demand is driven by health-conscious consumers and is supported by the expansion of glasshouse and sustainable farming systems [7].

## GSB as a worldwide pathogen and a protected-cropping biosecurity risk

**Fig. 1:**
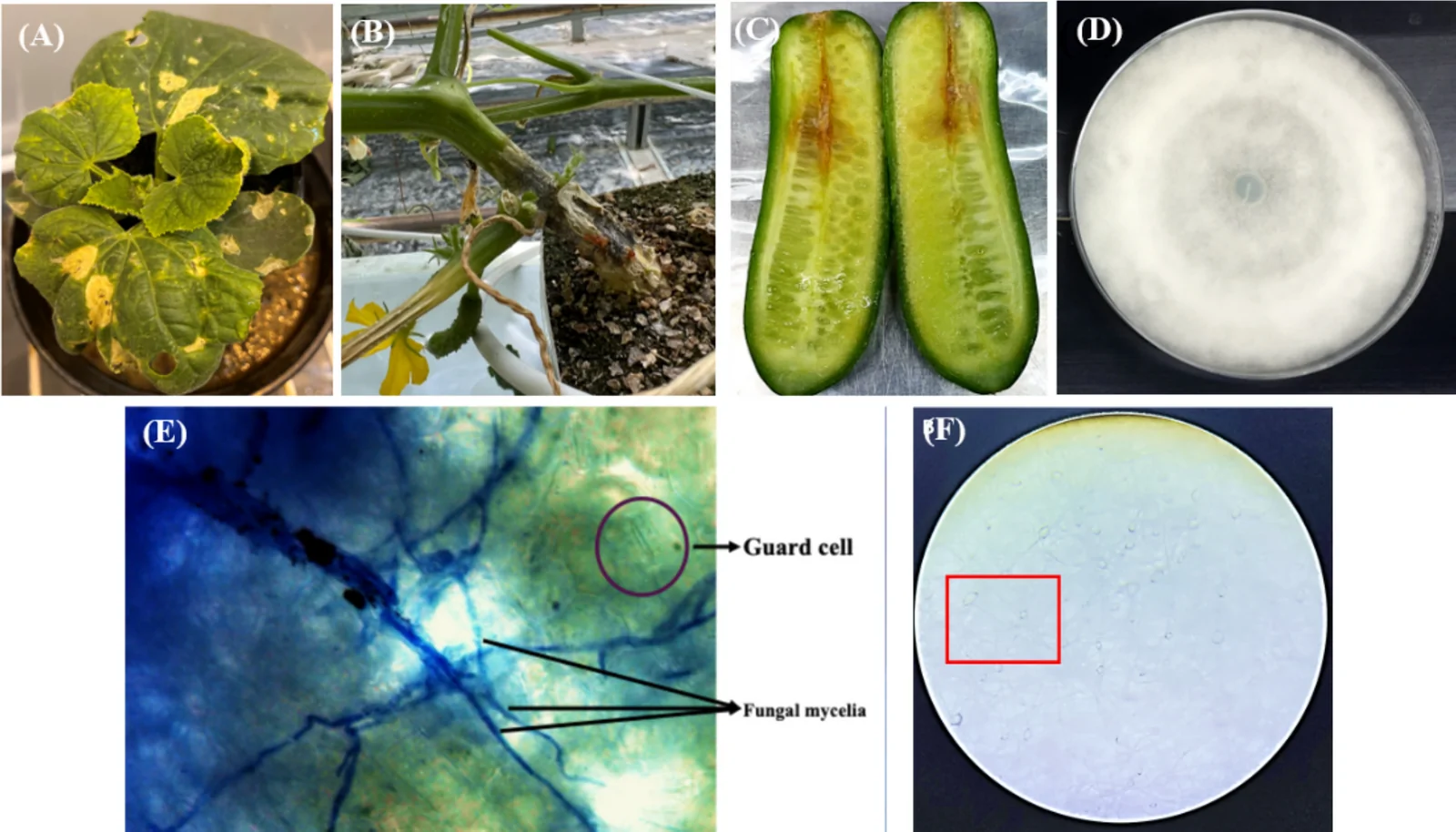
Gummy stem blight symptoms and fungal structures. **A** GSB infection at the seedling stage. **B** Stem infected with GSB. **C** Cucumber fruit infected with GSB. **D** Growth of GSB on ¼ PDA. **E** Mycelial structure visualised under a 20 × microscope after staining with lactophenol blue. **F** Pycnidia of GSB visualised on a ¼ PDA plate under a 10 × compound microscope

Diseases causing root or stem rot and plant wilt are among the most devastating for cucumber growers, causing up to 33% of plants to die prematurely [8]. The fungal disease commonly known as gummy stem blight (GSB), caused by *Stagonosporopsis* spp., is one of the most problematic cucumber diseases; infection can occur through cotyledons (Fig. 1A), hydathodes, injured trichomes and stem tissues (Fig. 1B), flowers, and fruit (Fig. 1C). The industry-facing difficulty is that fruit infection may remain asymptomatic for extended periods and become visible only weeks after harvest, when produce is moving through supply chains or reaching market shelves. This latency converts a biological disease problem into an industry challenge: growers require tools that can detect infection early enough to guide crop hygiene, harvest decisions, fungicide use, crop removal or replanting before commercial losses accumulate. GSB is caused by three *Stagonosporopsis* species that are morphologically indistinguishable but genetically distinct. These include *Stagonosporopsis citrulli*, *Stagonosporopsis cucurbitacearum* (synonymous with *Didymella bryoniae*; Fig. 1D) and *Stagonosporopsis caricae* [9]. *S. citrulli* and *S. cucurbitacearum* are closely related but distinct sister species that can be differentiated phylogenetically using a multilocus sequencing approach, whereas *S. caricae* constitutes a more basal lineage [9]. They belong to the phylum Ascomycota and the class Dothideomycetes [10]. Phylogenetic analysis of strains of *S. cucurbitacearum* and other members of Didymellaceae using RAPD and ITS sequences grouped the strains into four phylogenetic clusters: RAPD groups RG I, RG II, RG III and RG IV. Virulence assays on muskmelon seedlings showed that RG I isolates were highly virulent, with a disease severity of 71%, whereas RG II and RG IV isolates exhibited low virulence, with a disease severity of 4%, and RG III isolates were non-pathogenic. ITS sequencing confirmed that RG I and RG II were *S. cucurbitacearum* [11]. The optimum conditions for spore germination and hyphal development of GSB (Fig. 1E) include temperatures of 24–25 °C, humidity of 85%, and 1–10 h of persistent moisture [12]. The fungi causing GSB rapidly produce asexual fruiting bodies, pycnidia (Fig. 1F), and sexual fruiting bodies, pseudothecia, at later stages of disease. Conidia and ascospores contribute to within-crop and longer-distance dispersal, while pseudothecia can persist on crop debris and seeds (Fig. S1). These biological features are not only important for understanding GSB epidemiology; they define the sampling points that any industry-facing test must address. For cucumber glasshouses, symptom-based detection is too late for many management decisions because visible symptoms occur after the pathogen has already spread or latent fruit infection has entered the supply chain. Symptoms can also resemble those caused by other diseases, including *Phomopsis* black root rot and *Botrytis* rot. Therefore, the central diagnostic problem for GSB is not simply whether the pathogen can be detected in the laboratory but whether detection can be achieved early, cheaply and accurately enough under commercial production conditions to inform management. For this reason, this review treats GSB in cucumber as both a pathogen-biology problem and a technology-development problem. The following sections critically assess current detection technologies and draw on evidence from other pathosystems to identify practical pathways for pre-symptomatic GSB detection, resistance monitoring and disease control in glasshouse production systems.

## Early approaches for GSB detection

In the design and deployment of on-farm pathogen diagnostics, several factors must be considered, including specificity, sensitivity, the equipment, and training needed to run the analysis, and the time required to obtain a result. Owing to its simplicity and minimal equipment requirements, the most widely used detection method for seed-borne fungi such as GSB is a blotter test [13]. In this method, seeds are surface-sterilised and incubated on two layers of blotter paper in a humidity chamber in the dark for 10 days at 25–27 °C. Afterwards, seedlings showing symptoms (root discolouration or stem collapse) are cultured under a 12-h photoperiod for an additional 4–5 days so that pycnidia can form and spore morphology and size can be used to validate the presence of GSB [13]. However, this approach requires specialised morphological identification and cannot differentiate between morphologically identical *Stagonosporopsis* species (Table 1) [9], which requires laboratory-based genetic testing. Similarly, the morphologies of *S. cucurbitacearum*, *S. citrulli* and *Phoma* spp. are similar, making cross-genus identification difficult [14]. Furthermore, the blotter test requires considerable time before a positive diagnosis is obtained; in commercial environments, cucumbers with a 12-week growth period may already be dead or have spread the disease within the growing zone, limiting the utility of this approach. Thus, more specific tests with DNA-level detection limits were developed.

**Table 1:** The current Gummy Stem Blight detection methods based on genome- and proteome-guided approaches

	**Current detection methods**					
	**Advantage**	**Drawbacks**	**Efficacy**	**Limit of Detection**	**Time and cost**	**Ease of use and experience level**
Seed blotter test	• Easy to use • No specific primers	• Humidity chamber is needed • Not able to differentiate between *Stagonosporopsis* spp. or from other fungi with similar morphology	Helps in identifying *Stagonosporopsis* infected seeds	Unknown	• Time − 10–15 days • Cost- Medium	• Ease of use- Easy • Experience level- Expert in mycology • Equipment level- Standard
**DNA-based methods**						
RAPD (Random Amplified Polymorphic DNA), AFLP (Amplified Fragment Length Polymorphism)	• Utilization of single random primer • Amplification of several distinct DNA products	• Misclassification of isolates • Relies on short random primers which can lead to inaccurate priming and diagnosis • Laboratory equipment needed	Differentiates between *Stagonosporopsis* and *Phoma* species	0.1 ng of fungal DNA	• Time − 2–3 h • Cost- High	• Ease of use- Difficult • Experience level- Expert • Equipment level- Specialized
PCR-ELISA (Enzyme Linked Immunosorbent Assay)	• Addition of fluorescein and biotin on the RAPD primers • Colorimetric assay	• Large background signal • Troubleshooting for probe specificity and hybridization needed • Laboratory equipment needed	Differentiates between *S. cucurbitacearum* and *Phoma* species	10^4^ CFU/mL of virus	• Time − 3–24 h • Cost- High	• Ease of use- Difficult • Experience level- Expert • Equipment level- Specialized
Magnetic capture hybridization-Multiplex real-time PCR	• A TaqMan PCR test specific to the pathogen • 10 × more sensitive than direct real-time PCR	• Not all genotypes can be detected • Materials are quite expensive and require a molecular laboratory and equipment	Identify *S. citrulli* in both watermelon and melon seeds	4 fg of fungal DNA	• Time − 24–26 h • Cost- High	• Ease of use- Difficult • Experience level- Expert • Equipment level- Specialized
Multiplex conventional PCR	• Whole genome guided primer • Differentiate between the species of any plant tissue	• Generation of false positives a frequent occurrence • Expensive enzyme is required and specialized equipment is needed	Differentiate between *S.citrulli* and *S. cucurbitacearum*	0.01 ng/µL of fungal DNA	• Time − 2.5–4 h • Cost- High	• Ease of use- Difficult • Experience level- Expert • Equipment level- Specialized
Loop-mediated isothermal AMPlification (LAMP)	• 1,000 × more sensitive than conventional PCR • Employable in glasshouse and field conditions • Colorimetric assay can easily be monitored without specialized equipment	• The ability to differentiate between *Stagonosporopsis* species is documented for some species	• Isothermal and can be run with minimal set of equipment • Colorimetric	01.fg/µL of fungal DNA	• Time − 40–45 min • Cost- Medium	• Ease of use- Neutral • Experience level- Beginner • Equipment level- Standard

Designing DNA-based tests to be highly specific in the GSB pre-genomics era involved a significant effort in testing for cross-reactivity [15]. The sensitivity needed for pre-symptomatic detection was also difficult to parameterise for fungal pathogens, as their hyphal growth patterns make it complex to assess the number of infective units needed before visible disease occurs. For this reason, many fungal diagnostics rely on time since inoculation (e.g., *B. cinerea*) [16]. If we look to bacterial systems, however, we can get a better sense of the number of infective units (referred to as colony forming units; CFU) where studies have found that bacterial pathogens are typically asymptomatic in leaves between 10^5^–10^7^ CFU/cm^2^ of tomato [17]. Therefore, in fungal diseases, it is likely that we must get within the sensitivity range of a similar number of nuclei/copies of a diagnostic gene, or possibly even more sensitive to account for dilution effect in tissue extractions and assay inhibitors [18]. When considering the ease of use and field deployment of DNA-based diagnostics, a number of hurdles must be overcome, including large sample load in field conditions [19], cross-reactivity of primers while detecting multiple pathogens [20], reagent stability and difficulties in managing plant inhibitors in a field setting [21].

The earliest molecular diagnostic tool developed for GSB in leaves and fruit was based on random amplified polymorphic DNA (RAPD) analysis (Table 1). This technique uses a single random primer in a polymerase chain reaction (PCR), resulting in the amplification of several distinct DNA products [22]. Although simple to run, these assays rely on short random primers to create a fingerprint; the low annealing temperatures required can lead to inaccurate priming and diagnosis. Consequently, the RAPD approach is not always sufficiently specific for growers, as shown by [23], who found that the analysis misclassified three isolates from cucurbits compared with the secondary approach of amplified fragment length polymorphism (AFLP). Although the conversion of RAPD bands into SCAR markers increases reagent specificity and consistency in other systems (e.g., sensitivity down to 0.1 ng of DNA for *Pseudofabraea citricarpa* [24]), RAPD still exhibits low reproducibility and limited resolution in GSB [25].

A subsequent advance was the development of a PCR–enzyme-linked immunosorbent assay (PCR-ELISA; Table 1; [26]). In this process, PCR primers generated for RAPD fingerprinting of the RAPD Group I and II FP and RP regions were modified by adding fluorescein and biotin labels to the 5′ ends of the primers. Following amplification, ELISA was used to examine the products with a horseradish peroxidase-conjugated anti-fluorescein antibody and three substrates that yielded coloured products, one for each group and one for background. However, PCR-ELISA for general disease diagnosis is limited by high background signals and the extensive troubleshooting required to achieve probe specificity and hybridisation [27]. ELISA sensitivity and specificity depend on the assay and antibody type, which influence enzyme and substrate activity and can increase false-positive and false-negative results [28]. If these limitations can be overcome, GSB detection is comparable to that in other systems (e.g., *Odontoglossum* ringspot virus), with a sensitivity of approximately 10^4^ copies/mL [29]. Currently, the best use of this method is to differentiate between GSB and *Phoma* infection [30].

After nearly a decade, improvements were made upon RAPD fingerprinting with the advent of multiplex real-time PCR [31]. A similar assay was used successfully for the detection of *Fusarium oxysporum, F. tricinctum* and *F. solani* with sensitivity and detection limits of 1.46 × 10^3^, 7.71 × 10^3^ and 1.76 × 10^2^ copies/µL, respectively [32]. For *Stagonosporopsis* infecting cucurbits, magnetic capture hybridisation was coupled with multiplex real-time PCR, where the DNA template (a conserved 480 bp DNA fragment of *S. citrulli;* Table 1) was precisely concentrated using single-stranded DNA hybridisation capture probes covalently bound to magnetic particles. A TaqMan PCR test specific to the pathogen was then used to amplify the extracted DNA, creating a method 10 × more sensitive than direct real-time PCR that can successfully identify *S. citrulli* with a limit of detection of 4 fg of genomic DNA [33, 34]. However, not all genotypes could be detected by the assay; only isolates of *S. citrulli* (known as RG I) could be detected. Furthermore, the assay materials are expensive to purchase and require a molecular laboratory, making them inaccessible for most growers.

## Post-genomic detection of GSB

In a significant advancement for the field of GSB detection and study, the genome of *Stagonosporopsis cucurbitacearum* was assembled for the strain Zq-1, whose resulting genome size was 35.28 Mbp and had 9,844 predicted protein-coding genes, including 756 secretory proteins with 278 secreted proteins, which included cell wall-degrading enzymes and putative effector proteins that may be involved in host colonisation [35]. When pathogens attack plants, they produce small secreted ‘effector’ proteins that, following release from the pathogen, can move into the host tissues and target/manipulate host protein and nucleic acid function [36]. These effector molecules can also remain in the plant apoplast, where they enzymatically degrade plant cell walls or can inhibit plant proteolytic activity [37] or produce reactive oxygen species (ROS) [38]. In response to effector proteins, some plants have evolved receptor proteins that bind the effector molecule and induce the production of hypersensitive molecules and programmed cell death [39]. In cases where the pathogen is biotrophic, this localised cell death stops the development of disease.

For necrotrophic pathogens such as GSB, this system is leveraged by the pathogen during disease expression to generate the dead tissue from which they feed. However, if the production of these effector proteins could be stopped or reduced, it would interrupt disease progression [40]. The *S. cucurbitacearum* ScAA9 effector protein was found to act as both a virulence factor and a PAMP for altering plant immune responses, whereby elevated expression attenuated virulence in watermelon plants [41]. The genome of another *S. cucurbitacearum* strain, DBTL4 isolated from diseased watermelon plants, was 34.08 Mb, with a prediction of 10,748 protein-coding genes, of which 812 were identified as putative secretory proteins with 113 potential effectors [42, 43]. Another study presented the genome sequence of *Stagonosporopsis vannaccii*, identified from soybean with a genome size of 41.3 Mb having 11,237 genes having secreted effectors and secondary metabolite clusters [44]. Therefore, there is quite a range of intra- and inter-generic differences in *Stagonosporopsis* that could be used to modify or tune detection methods. During this time, *Stagonosporopsis* was also found to produce diverse small molecules that could be used in the detection of the pathogen. These included alkaloids such as stagonoculiepine and stagonoculiazin from *Stagonosporopsis oculihominis* [45], 4-hydroxy-2-pyridone isolated from *S. cucurbitacearum* [46], and stagonosporopsin C from *S. oculihominis* [47].

Using comparative genomics, a GSB species-specific PCR method was developed to differentiate among *Stagonosporopsis* species using a multiplex conventional PCR assay (Table 1). The assay uses three primer sets: Db01, which amplifies a 356–374 bp fragment only from *S. citrulli* isolates; Db06, which amplifies a 283–289 bp fragment in *S. citrulli* and a 286 bp fragment in *S. cucurbitacearum*; and Db05, which amplifies a 216–224 bp fragment in all three species [48]. Species identity is then inferred from the banding pattern after DNA gel electrophoresis. However, because only one band differentiates *S. citrulli* from *S. cucurbitacearum*, failed amplification of the Db01 fragment can generate false negatives, and the assay has been reported to be unreliable at low pathogen titre levels [49]. Improvements in primer design and amplification chemistry may increase sensitivity, as reported for *Fusarium oxysporum* detection in quarantine-exported cacti, where 0.01 ng/µL of genomic DNA could be detected [50]. With more *Stagonosporopsis* genomes becoming available, this approach could also be adapted for strain-level identification, as demonstrated for *Erwinia amylovora* [51]. However, the current format remains moderately resource-intensive because it still relies on gel electrophoresis for interpretation [52].

The most rapid, sensitive and visual form of GSB detection is the loop-mediated isothermal amplification (LAMP) assay (Table 1) [53]. LAMP uses *Bst* DNA polymerase, strand displacement and four to six primers targeting distinct regions of the DNA template, making the protocol highly specific [54]. The reaction runs isothermally, so it requires less equipment than conventional PCR and can be read visually using colorimetric indicators [55]. For GSB, LAMP has shown 1,000-fold higher sensitivity than conventional PCR, can be completed within 40–45 min, and has been used for early disease detection under glasshouse and field conditions [56]. A recent watermelon assay specific for *S. citrulli*, the SCIT850 LAMP assay, displayed analytical sensitivity of 1 pg of genomic DNA by targeting a single-copy SCAR sequence shared across *Stagonosporopsis* RG groups and the *MAT1-1* locus [57]. Similar sensitivity has been achieved in other pathosystems, including *Hemileia vastatrix*, where LAMP detected as little as 1 pg of DNA within 1 h [58], whereas other systems have reached limits as low as 0.1 fg/µL of genomic DNA [59]. These results indicate strong potential for pre-symptomatic GSB detection, but current GSB LAMP assays still require further field validation, improved species discrimination and careful control of visual indicators that can inhibit polymerase activity or increase the risk of misleading results [60].

## GSB treatment and known modes of fungicide resistance

Following detection, growers can treat GSB with a range of fungicides, although this option is constrained by cucumber harvest schedules because crops are often picked on a 24-h rotation and few registered products have sufficiently short withholding periods. Reports of fungicide resistance are also increasing, as reviewed in detail in [61]. Resistance can arise through conserved point mutations or through more variable changes within target genes (Table 2). An example is resistance to the quinone outside inhibitor (QoI) fungicide azoxystrobin, one of the earliest synthetic fungicides used for GSB control [67]. Resistance to azoxystrobin in *S. cucurbitacearum* is often conferred by a point mutation in the mitochondrial cytochrome B gene (CYTB), which alters protein structure and prevents fungicide binding [68], while in *S. citrulli,* resistance is associated with a nucleotide substitution at amino acid position 143. For cucumber growers, fungicide-resistance information is only valuable if it is returned quickly enough to influence spray choice, rotation strategy, crop hygiene and block-level disease containment. Conventional culture-based fungicide sensitivity testing requires isolation of the causal agent, growth in pure culture, laboratory infrastructure and time frames that are poorly aligned with rapid cucumber production cycles. Despite the continuing need for chemical fungicides in production systems, their off-target effects and the rise of resistance are increasing demand for alternative control methods.

**Table 2:** Different types of fungicides for preventing Gummy Stem Blight in cucurbits

**Fungicide class**	**Fungicides**	**Molecular mechanism**	**Resistance development**	**Species with resistance reported**		**Location**
Quinone outside Inhibitor (QoI)	Azoxystrobin	Single site acting fungicide at *cytb*	Resistance develops by one point mutation in *cytb*	Detected in *S.citrulli*		Resistance development cases reported in 2001 and 2002 in Georgia and South Carolina [62]
Succinate dehydrogenase inhibiting (SDHI)	Boscalid	Targets succinate dehydrogenase complex in cellular respiratory change	Single amino acid substitution in one or more subunits of SDHB	Genotypes of *S. cucurbitacearum* and in *S. citrulli*		Resistance development cases in 2007 in Georgia and South Carolina [10, 63]
Demethylation inhibitor (DMI)	Tebuconazole	Targets CYP51	Low and moderate resistance found after point mutation in CYP or in the promoter region of Cyp51 leading to over-expression		In *S.citrulli* and *S.caricae*	Reported in watermelon fields in Zhejiang Province, China [64], Georgia and South Carolina [65, 66]

## Breeding for disease resistance: the ultimate disease control

A range of classical breeding techniques have been used in recent years to identify GSB resistance regions of different host plants, as described in [61]. In melon, a cross between susceptible cultivated melon and the more resistant wild relative *Citrullus amarus* identified a major quantitative trait locus (QTL) named Qgsb8.1, which confers resistance to GSB caused by *S. cucurbitacearum* strain JS002 in the watermelon line PI189225 [69]. Further bulk segregant analysis (BSA), relying on single-nucleotide polymorphisms (SNPs), was then used to pinpoint this QTL in a population of 211 hybrid plants, with the resistant region spanning approximately 5.7 Mb [69]. Another GSB QTL study in melon identified a receptor-like kinase family protein named a Wall-associated kinase (WAK) associated with GSB resistance: on chromosome 7, in a 140 kb physical interval, a WAK gene named MELO3C010430 was annotated with several nonsynonymous SNPs and a gene expression profile consistent with resistance to GSB infection [70]. Discoveries such as these are crucial for developing new, disease-resistant varieties through marker-assisted selection (Table 3). As mentioned in [61], a study of 160 F_9_ cucumber recombinant inbred lines obtained from a cross between a GSB-resistant accession (PI 183967) and a GSB-susceptible accession (PI 189931) led to the identification of five GSB-resistant QTLs named-*gsb-s1.1, gsb-s2.1, gsb-s6.1, gsb-s6.2* and *gsb-s6.3* [71], complementing previous work identifying cucumber QTLs associated with GSB resistance [72].

**Table 3:** Advanced protective measures to limit Gummy Stem Blight establishment and spread based on pathogen genome- and proteome-guided approaches

**Advanced Protective Measures**				
		**Proposed approach**	**Advantage**	**Disadvantage**
**Fungicides**	Genome guided	LAMP (Loop mediated isothermal AMPlification) based selection of fungicides with respect to the resistant mutant isolates or point mutations associated with fungicide resistance	Will guide the use of specific and targeted fungicides Sensitive enough to identify single point-mutations	Limited knowledge exists on the molecular basis of fungicide resistance across different species
	Proteomic guided	Based on fungal effector proteins Use of dsiRNA (Dicer substrate small interfering RNA) targeting early stages of pathogen development	Species- Specific RNA interference Development of fungistatic dsiRNA to control disease development in a specific and environmentally friendly manner	Efficacy specifically dependent on competency of RNA uptake by the pathogen
**Resistant varieties**	Genome guided	Based on QTL (Quantitative Trait Locus) CRISPR/Cas precise editing at a predetermined location which helps in gene deletion, correction or insertion	Development of disease-resistant varieties through marker-assisted selection Development of completely resistant varieties to pathogen New genotype can be developed in short time	Using marker assisted approach can also bring unwanted linked genes which can reduce crop efficiency Gene editing of one gene is usually insufficient
	Proteomic guided	Receptors of effector proteins in the plant and transcription factors	More specific based on effector-controlled disease progression	Limited information in the area of target receptor proteins

Beyond QTL approaches, genomics and transcriptomics have identified specific plant genes and pathways associated with disease progression. For example, transcription factors modulate gene expression involved in plant growth and pathogen responses [73]. Among these, WRKY transcription factors participate in both pattern-triggered immunity (PTI) and effector-triggered immunity (ETI) and regulate the biosynthesis of defense compounds [74]. Five WRKY DNA-binding proteins have recently been implicated in GSB defense responses and defense-compound production in pumpkin [75]. AP2/ERF transcription factors are known to contribute to development and responses to both abiotic and biotic stress; after GSB infection, 15 genes were differentially expressed and may contribute to stress tolerance and signal transduction [75]. In the same study, 13 bHLH transcription factors were also implicated in the immune response to GSB. TGA transcription factors and genes encoding receptor-like cytoplasmic kinases showed differential expression in response to *S. cucurbitacearum* in melon [76]. Genes encoding the chaperone protein DnaJ, metallochaperones, aspartic proteases and MYB transcription factors were more highly expressed in the resistant line [77].

**Fig. 2:**
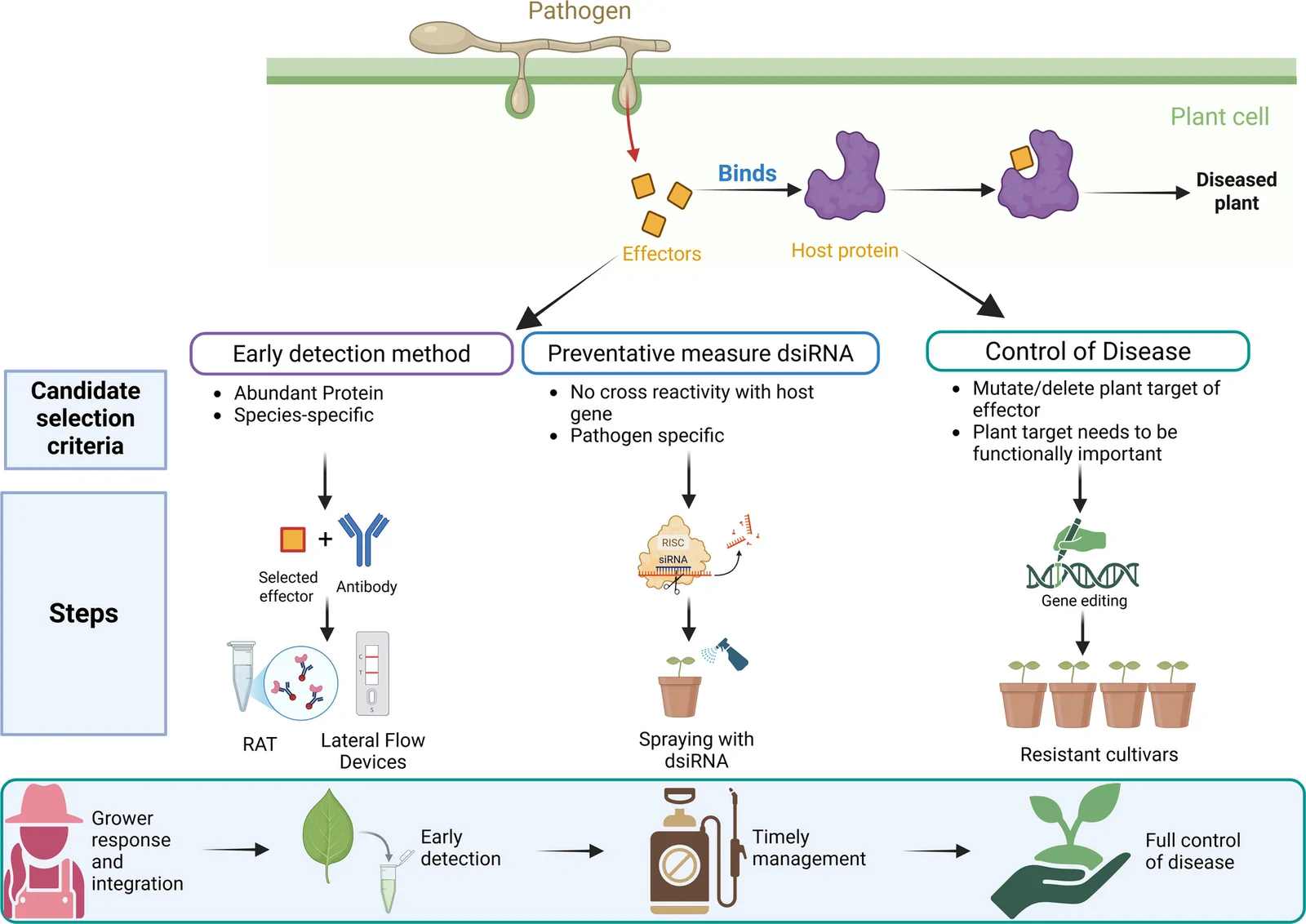
Functional understanding of GSB effector proteins as a bridge between disease biology, pre-symptomatic detection and sustainable disease control. Effector proteins released during early infection can interact with host proteins and alter plant immune signalling. Detecting these proteins could provide an early, grower-usable signal of active GSB infection, while functional knowledge of the same effectors could inform dsiRNA targets, resistant cultivar development or editing of host susceptibility factors. The figure therefore links the core GSB review thread to a cucumber glasshouse adoption pathway: identify effectors, validate them during infection, convert them into detection targets, and use that knowledge to guide control or breeding decisions. Figure prepared with BioRender

Targets and receptors of GSB effector proteins can also be used to develop resistant varieties (Fig. 2; Table 3). In melon, an R gene located on chromosome 9 that displays sequence polymorphisms between resistant and susceptible lines was linked to resistance against GSB [78]. In another study, brassinosteroid insensitive 1-associated receptor kinase 1 (BAK1) was found to act as part of a complex that inhibits R-gene expression in effector-triggered immunity [79], suggesting that loss of these kinases could improve host resistance. In support of this, most cucumber-encoded BAK1 genes (*CsBAK1*) are downregulated in GSB-resistant lines after infection, while two genes (*CsBAK1-8* and *CsBAK1-14*) show significantly higher expression in susceptible lines than in resistant lines [80].

To date, none of these QTLs or specific genes have been introgressed into commercially available cucumber cultivars to provide durable GSB resistance. Nevertheless, these studies identify the kinds of loci that could support traditional breeding of disease-resistant cultivars (Table 3). Traditional breeding remains time-consuming and can introduce linkage drag that negatively affects agronomically important traits such as yield. If specific host genes that mediate partial or complete resistance can be validated, particularly genes involved in effector-controlled disease progression, then additional technologies could be used to accelerate cultivar development.

## The future of grower-friendly GSB detection and control: a staged framework for technology development and application

For adoption in cucumber glasshouses, future DNA-based GSB diagnostics should be evaluated against practical use criteria rather than analytical sensitivity alone. A deployable assay must tolerate crude plant extracts, detect pathogen DNA before symptom development, distinguish relevant *Stagonosporopsis* species where this changes management, produce an interpretable result within a same-day decision window, and integrate with crop records so that positive results trigger defined management actions. However, those management responses also need further development because growers require tools that can better suppress and eradicate GSB from production systems. Commercially available cucumber cultivars with resistance to GSB are lacking, so growers remain highly reliant on fungicides; however, as in other pathosystems, repeated fungicide use has selected for resistant pathogen populations. Improved understanding of the molecular basis of GSB colonisation should therefore support not only pathogen detection but also new disease-management strategies. The following staged framework moves from near-term, grower-deployable diagnostics to longer-term control strategies that integrate genomics, proteomics, resistance monitoring and host improvement. By organising these opportunities as sequential development stages, the review links pathogen biology to practical decision points where new tools could change cucumber glasshouse management.

### Stage 1: Species-specific, field-applicable DNA detection for cucumber glasshouses

Although a verified species-specific LAMP assay for GSB has yet to be developed, such assays have been developed for other pathogens, including *Colletotrichum nymphaeae* and *C. acutatum* [81] and four *Fusarium* species, with detection limits as low as 100 pg/µL [82]. LAMP often fails to distinguish between species because primers are designed for intergenic regions of the genome. However, recent work shows that the use of species-specific genes can overcome this limitation. For example, *Fusarium graminearum*, a major producer of the mycotoxins DON and zearalenone in brewing cereals [83], was specifically identified using a class II hydrophobin gene (*hyd5*) after comparative genomics showed that this gene can distinguish harmful species from closely related endophytes [84]. Therefore, as highlighted in the genome descriptions of different *Stagonosporopsis* species above, species-specific genes could be identified and used to distinguish *S. cucurbitacearum* from other *Stagonosporopsis* species in LAMP ‘v2.0’ assays (Table 4).

**Table 4:** Future Gummy Stem Blight detection methods based on genome- and proteome-guided approaches

	**Detection methods with future potential**			
	**Improved efficacy**	**Improvement in time and cost efficiency**	**Potential for improved ease of use**	**Implementation barrier**
LAMP v2.0	• Species-specific detection for all species of *Stagonosporopsis* from tissue samples also except from pure DNA • Low detection limits up to 1 pg/ul of pure DNA with 20–26 copies/ul (if LAMP conserved region is targeted for amplification) of *Stagonosporopsis*	• Target: 30 min • Advantage: Time savings of up to 15 min • Cost- High	• Can be done on field • No specific equipment only water bath is needed • Not much technical skills are needed	• Difficulty in primer designing • Due to cross and carry-over contamination false positives • Subjective colour change
CRISPR (Clustered Regularly Interspaced Short Palindromic Repeat)	• Specificity down to one single nucleotide difference • Operates at 37^0^C • Time efficient • Crude leaf extract for DNA samples can be used • With low detection limits of 10 pg/µL in genomic and plant DNA samples	• Target- 5–30 min • Advantage- Time savings up to 30 min • Cost- Medium	• On-field detection • No specific. equipment and technical skill are needed	• Designing of gRNA • Environmental factors pH and temperature
RAT (Rapid Antigen Test)	• Antigen–antibody test • Colorimetric assay • Leveraging the information of effector proteins used by GSB to elicit disease • Based on determination of specific markers of the pathogen • It can detect up to 500 CFU/mL of bacteria	• Target- Detection 3–10 min • Advantage- Time saving up to 30–60 min • Cost- Low	• Specific • Sensitive • Easy to use for the growers	• Crude extract can add inhibitors • False positives

To support comparative genomic analysis, a rapid diagnostic system for watermelon, the SCIT850 LAMP assay, was developed [57]. This method still requires field validation and testing alongside a second species-specific LAMP assay. Therefore, a global pan-genome sequencing effort within and between *Stagonosporopsis* species is needed, using a genome of *S. cucurbitacearum*, such as strain DBTL4, as a basis for comparison [43]. LAMP v2.0 also faces implementation barriers, including primer-design difficulties: the use of six primers can lead to cross-reactions and false-positive results [85]. It can also be difficult to identify a target region that is sufficiently specific and long enough to accommodate all primers without hindering efficient amplification [86]. From a practical grower perspective, reaction-vessel design also requires attention because carry-over contamination between small tubes is highly likely [87]. In addition, a clear positive/negative colour change is needed because the colour shifts in current LAMP assays can be subjective and difficult to interpret [88].

One of the most recent advances in pathogen detection, yet to be applied to GSB, is the use of clustered regularly interspaced short palindromic repeat (CRISPR)-associated proteins to develop novel biosensors that use isothermal amplification and simple reporting formats such as lateral-flow strips (Table 4) [89]. This procedure uses CRISPR arrays and Cas operons in three steps: adaptation, synthesis of CRISPR RNA, and target interference [90]. It can distinguish a single-nucleotide difference and operates at 37 °C [91]. In a study detecting the fungus *Magnaporthe oryzae* pathotype *triticum* (MoT), field detection was achieved using Cas12a, recombinase polymerase amplification (RPA) and a nucleic acid lateral-flow immunoassay (NALFIA) on a paper-based platform, with detection and quantification completed within 5–30 min [89]. A system was also established to identify *Diaporthe aspalathi* and *D. caulivora* in soybean using RPA-CRISPR/Cas12. The total time from DNA extraction to detection was less than 60 min using lateral-flow strips and portable blue flashlights [92]. These examples, however, still rely on relatively pure inputs. To improve accessibility for growers, [93] adapted a CRISPR/Cas system for the simultaneous detection of five apple viruses using crude leaf extracts, removing the need for purified DNA. In this method, DNA was crudely lysed from cells with a hand-held homogeniser, and the reaction was incubated at body temperature, producing results in less than 1 h from sample collection. Similarly, detection of *Verticillium dahliae* in soil using a combined LAMP and CRISPR/Cas12a assay was completed in less than 90 min by fluorescence-based detection and in less than 120 min by lateral flow using crude DNA extracts with detection limits of 10 or 1 fg/µL of genomic DNA [94]. Therefore, the underlying technology needed to advance GSB detection using CRISPR/Cas is available. However, beyond assay design, limits of detection during the earliest stages of pathogenesis must be addressed [95], partly through careful gRNA design [96]. Environmental factors such as temperature and pH also affect system stability; therefore, appropriate buffers and assay conditions must be established for effective field deployment [97].

Within this first stage, the immediate roadmap for adapting and improving GSB DNA- or RNA-based detection requires expanded isolate sampling for comparative genomics. This will enable the identification of conserved and species-discriminating loci and the design of LAMP or CRISPR/Cas assays specific to these regions. Further work is also needed to validate these assays against symptomatic and asymptomatic cucumber tissues under glasshouse conditions. Finally, the results could inform subsequent management steps, including treatment, sanitation and crop-removal thresholds.

### Stage 2: leveraging early GSB proteomic signalling for pre-symptomatic detection

Another novel approach to detecting GSB could leverage the expression and deployment of pathogen effectors during host colonisation (Fig. 2; Table 4). From a diagnostic perspective, these proteins are attractive because they are often species-specific and can be produced in thousands of copies per pathogen cell during early infection, whereas DNA targets may occur at only one or two copies per cell. Consequently, very little pathogen tissue may be needed before detection becomes possible. Although hundreds of predicted effectors in the *S. cucurbitacearum* genome have yet to be evaluated as diagnostic targets [43], related systems demonstrate the potential of this approach. For example, an anti-SDE1 antibody targeting a Sec-delivered effector of *Candidatus Liberibacter asiaticus* enabled pathogen detection with greater sensitivity than traditional qPCR approaches [98]. Early detection of pathogen effector proteins could therefore support the development of a rapid antigen-style test (RAT) for growers to detect active GSB infection (Fig. 2; Table 4). Such a method could be specific, sensitive and easy for growers to use. However, it also poses challenges because crude plant-tissue extracts may contain a wide range of microbes or release inhibitors during cell lysis, affecting molecular analysis and potentially producing false-positive or false-negative results [99]. Before this approach becomes feasible, further transcriptomic studies of *Stagonosporopsis* infection across different hosts are needed to identify disease-specific markers that are amenable to rapid detection.

### Stage 3: genome-guided fungicide selection and resistance-aware decision support

Given the difficulty and timelines of current testing for fungicide resistance in GSB-affected crops, the technology-development roadmap for GSB requires resistance diagnostics that move from retrospective explanation to prospective decision support. Targeted tests for known resistance-associated mutations could be used alongside pathogen-detection assays to indicate whether a detected GSB population is likely to respond to a particular fungicide group. Such tools would not replace integrated management but would help growers reduce unnecessary sprays, choose more appropriate rotations or mixtures, and slow the development of fungicide resistance in pathogen populations. Although not currently used for GSB detection, molecular- marker tests for basic resistance mechanisms (e.g., point mutations in specific genes) could be used to monitor field populations and guide fungicide applications in real time, as has been achieved in other pathosystems (Table 3). For example, LAMP assays have been developed to differentiate the F_167_Y mutation in *F. graminearum*, which is associated with resistance to carbendazim fungicides [100]. Within the GSB system, genomic analyses could target genes associated with fungicide resistance in other fungal models (e.g., histidine substitutions in the iron–sulfur subunit B of succinate dehydrogenase or point mutations in cytochrome P450 genes) and support the design of new LAMP-based tests. Using this approach, if a boscalid-resistant GSB population was identified, growers could switch to pyrazole-carboxamide or pyridinyl-ethyl benzamide, two SDHIs that have been evaluated for GSB control in watermelon [101] and have had few reports of resistance in *S. cucurbitacearum* over the past decade [102]. A fungicide-resistance assay could also guide the use of co-formulations (Table 2). For example, despite known resistance of GSB genotypes to individual fungicides, fungicide co-formulations have controlled more than 83% of disease incidence in the field, outperforming individual agents [103]. However, even if fungicide diagnostics become sensitive enough to identify single point mutations associated with resistance, growers will still need guidance on selecting alternative control measures. This is particularly important if they choose another single fungicide whose efficacy could be rapidly compromised by point mutations in the pathogen. Instead, we advocate using either multiple fungicides in rotation or fungicides that target several fungal proteins simultaneously, thereby reducing resistance risk. Additionally, these genomic DNA-based tests do not account for the expression of fungicide-detoxification enzymes, which can confound fungicide selection [104]. Therefore, multi-pronged detection platforms should be developed to better support fungicide selection and reduce fungicide overuse globally.

### Stage 4: proteomic-guided and RNAi-enabled fungistatic control options

As an alternative to traditional chemical fungicides, the development of pathogen-specific fungistatic compounds or treatments that interrupt plant–microbe signalling is gaining traction (Table 3). One versatile approach is the use of Dicer-substrate small interfering RNA (dsiRNA) to interrupt the early stages of pathogen development and potentially control GSB [105]. This approach has been used successfully against *Podosphaera xanthii*, which causes cucurbit powdery mildew. This pathogen suppresses chitin-induced plant immunity through the deployment of the effector genes *PxCDA* and *PxEWCAs*, whose products act as chitin deacetylases and promote chitin degradation, respectively. In this study, cotyledons infiltrated with dsiRNA showed an 80% reduction in fungal biomass and 50% suppression of effector expression [106]. In another study, species-specific RNA interference treatments reduced fungal infection and disease symptoms caused by *Sclerotinia sclerotiorum* [107]. Similarly, dsiRNA-mediated inhibition of *Botrytis cinerea* effectors reduced lesion size [107]. These findings demonstrate the adaptability and flexibility of this technology for protecting plants against devastating fungal pathogens. However, applying this approach to GSB control in cucumber will require further research into the identity and function of effector proteins—work that has begun in other host plants of this pathogen (Table 3). During pumpkin infection by *S. cucurbitacearum*, transcriptomic analysis identified a broad panel of differentially expressed genes, including 113 putative effectors [43]. The most effective fungal targets for dsiRNA treatment should be genes that are expressed during early infection and are highly specific to the target pathogen, as these are likely to be most effective at limiting fungal growth during critical early stages [108]. Although many options therefore exist for spray-induced gene silencing, candidate effector proteins must be functionally characterised before the best targets can be selected for the development of fungistatic compounds such as dsiRNA. dsiRNA design will also be critical because efficacy in other systems depends on the within-transcript target position, dsRNA architecture, functional target class and the pathogen’s capacity for RNA uptake [109]. The cost of this approach must also decrease substantially before it becomes economically viable for most growers. Thus, although early evidence indicates that GSB effector proteins are active and important during disease progression [41, 43, 69], further fundamental research is needed before a grower-ready application can be developed.

### Stage 5: advanced breeding techniques to develop GSB-resistant cucumber varieties for protected cropping

Genetic tools and broader genome-editing approaches are rapidly reducing the time required to develop new cultivars from decades to years or months [110, 111]. Over the past 20 years, genetically modified organisms (GMOs), produced using recombinant DNA technologies that introduce endogenous or heterologous genes into a host plant, typically under the control of a specific promoter, have become more widely used [112]. This methodology has been used to produce 525 transgenic events across 32 crop and flower species in 26 countries [113]. Transformation is commonly achieved through *Agrobacterium*-mediated methods or biolistics [114, 115]. However, the use of this technology remains limited in some contexts and lacks broad social licences. Newer non-GMO approaches, such as CRISPR/Cas-mediated genome editing, have therefore been developed [116]. This technique enables precise cuts in an organism’s DNA at predetermined locations using site-directed nucleases and a range of DNA-binding systems. After a double-strand break, the cell’s own DNA-repair mechanisms recognise and repair the damage through non-homologous end joining or homology-directed repair, leading to gene disruption or deletion, gene correction or modification, or DNA insertion [117]. We therefore advocate leveraging the knowledge described above to edit plant host proteins manipulated by GSB effectors (e.g., R proteins or WRKY transcription factors) and thereby limit disease progression (Fig. 2; Table 3). Through a gene-editing ‘stacking’ approach, multiple gene targets could be edited to reduce the probability that *Stagonosporopsis* would overcome these changes.

## Conclusion: a GSB review translated into a cucumber glasshouse adoption roadmap

GSB remains a cucurbit disease problem, but in cucumber glasshouse systems, it is also a technology-adoption problem. The same biological features that make *Stagonosporopsis* difficult to manage—latent infection, symptom overlap with other diseases, morphologically similar species, seedborne and debris-borne survival, and rapid spread under humid conditions—also define the requirements for the next generation of management tools. This review therefore connects core GSB biology to a staged pathway for industry translation: species-specific diagnostics, protein-based early detection, resistance-aware fungicide decisions, targeted molecular control and durable resistant cucumber cultivars. The central challenge is to convert genomic and proteomic knowledge into tools that are fast, affordable, robust in crude samples, interpretable by growers and compatible with commercial glasshouse workflows. Framing GSB research in this way does not reduce the importance of fundamental biology; rather, it makes clear which biological questions most directly support earlier detection, better control decisions and more sustainable cucumber production.

## Supplementary Information

Supplementary Material 1.

## Data Availability

Not applicable.
