# Supplementary material for: Translating pathogen biology into diagnostic and management strategies for gummy stem blight in cucumber production
Source: Crop Health. 2026 Nov 13;4(1):27. doi: 10.1007/s44297-026-00091-6 (PMC13759912; doi:10.1007/s44297-026-00091-6)
Supplement: Supplementary file 1 [file 44297_2026_91_MOESM1_ESM.docx]

**Supplementary Figure 1. Disease cycle of Gummy Stem Blight (*S.cucurbitacearum*) in cucumber.**
